# Myeloid sarcoma: a report of four cases at unusual sites

**DOI:** 10.3205/000244

**Published:** 2017-02-09

**Authors:** Fouzia Siraj, Manveen Kaur, Varsha Dalal, Ashima Khanna, Afaq Ahmad Khan

**Affiliations:** 1 National Institute of Pathology, ICMR, Safdarjung Hospital Campus, New Delhi, India; 2 JLNM Hospital, Srinagar, India

**Keywords:** myeloid sarcoma, granulocytic sarcoma, extramedullary, acute leukemia

## Abstract

**Background:** Myeloid sarcoma (MS) or granulocytic sarcoma is a rare tumor consisting of myeloid blasts with or without maturation occurring at anatomic sites other than the bone marrow. MS can involve any organ system but shows a predilection for skin, bone, and soft tissues of head and neck region.

**Case report:** We report four cases of MS occurring at unusual sites, out of which three were de novo and one was associated with acute myeloid leukemia (AML).

**Conclusion:** Although MS is associated with AML, it can rarely present without any existent hematologic disease. Differential diagnosis of a soft tissue mass should include MS even in the absence of leukemia. Establishment of the correct diagnosis depends on morphologic, histochemical, and immunohistochemical examination.

## Zusammenfassung

**Hintergrund:** Das myeloide Sarkom (MS) oder das granulozytäre Sarkom ist ein seltener Tumor, der aus myeloiden Blasten mit oder ohne Reifung besteht, die an anderen anatomischen Stellen als dem Knochenmark auftreten. MS kann jedes Organsystem involvieren, zeigt aber eine Vorliebe für Haut-, Knochen- und Weichgewebe der Kopf- und Halsregion.

**Bericht:** Wir berichten über vier Fälle von MS, die an ungewöhnlichen Stellen auftraten, von denen drei de novo waren und eine mit akuter myeloischer Leukämie (AML) assoziiert war.

**Schlussfolgerung:** Obwohl MS mit AML assoziiert ist, kann es selten ohne vorhandene hämatologische Erkrankung vorkommen. Die differentielle Diagnose einer Weichgewebemasse sollte MS sogar in Abwesenheit von Leukämie umfassen. Die Ermittlung der korrekten Diagnose hängt von der morphologischen, histochemischen und immunhistochemischen Untersuchung ab.

## Introduction

Myeloid sarcoma (MS), also known as extramedullary acute myeloid leukemia (AML), granulocytic sarcoma (GS) or chloroma, is a tumor composed of myeloid cells occurring at an extramedullary site [1], [2]. The most common sites of involvement include skin, bone, lymph node and soft tissues [2]. MS predominantly consists of cells of the myeloid lineage in varying stages of maturation, from myeloblasts to promyelocytes to neutrophils, that result in effacement of the tissue architecture. It can occur in four clinical situations: in patients with AML as a localized tissue mass, as blast crisis in patients with chronic myeloid leukemia (CML) or leukemic transformation in myelodysplastic syndrome (MDS), before AML and as an isolated neoplasm without evidence of AML.

We report four cases of MS to highlight the unusual clinical presentation of this entity and the diagnostic dilemma it can pose (Table 1 (Tab. 1)).

## Case description

### Case 1

**Figure 1:**
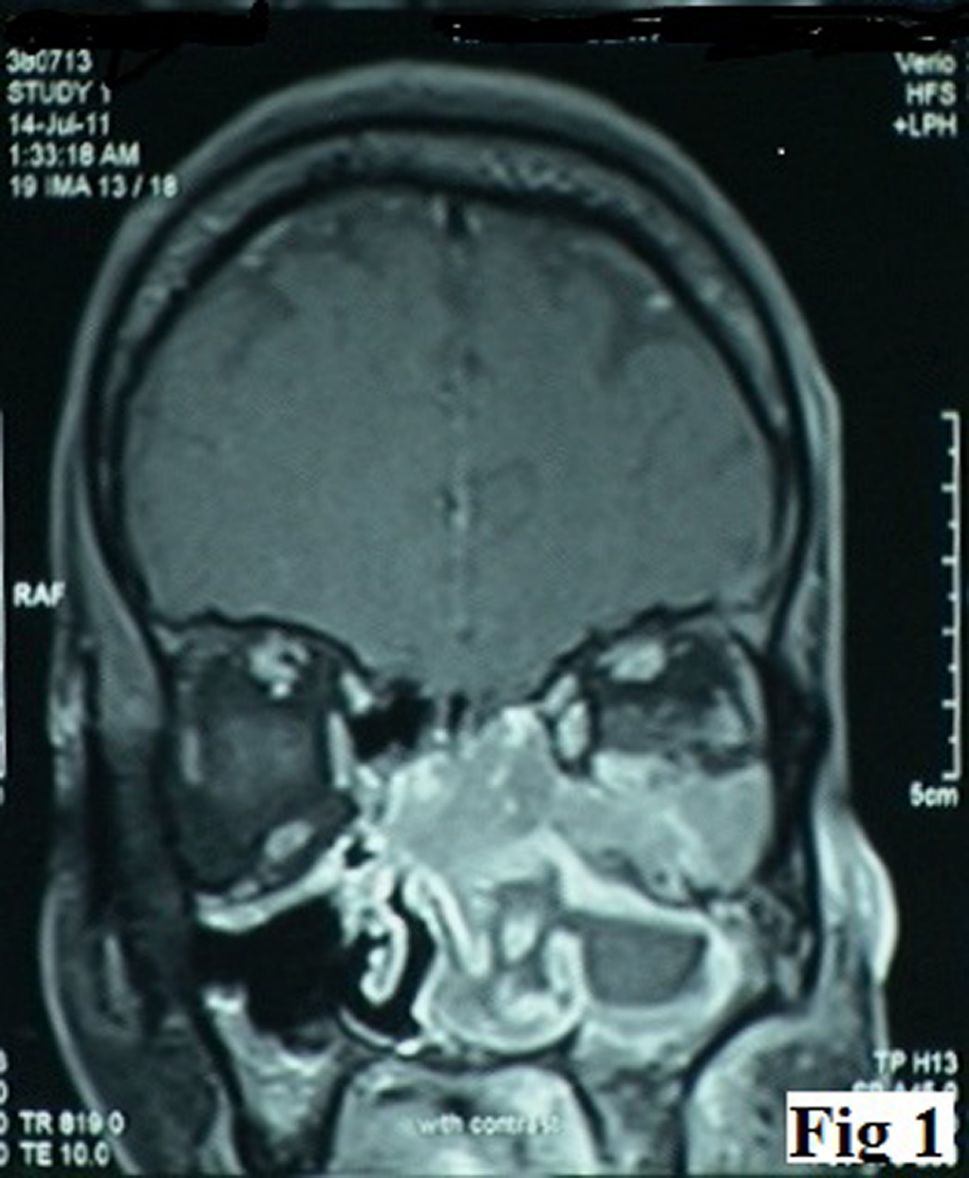
CT scan showing an intranasal mass involving the bilateral ethmoid sinuses with the destruction of the ethmoid septae and the medial wall of the left orbit.

**Figure 2:**
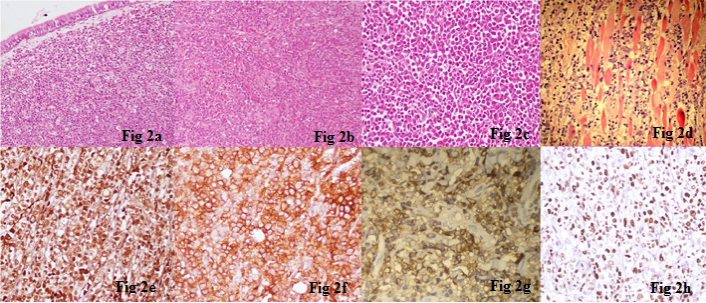
a: Photomicrograph showing sheets of immature cells admixed with interspersed mature inflammatory cells including eosinophils and their precursors (H&E, x 200). b: Large atypical cells with vesicular nuclei, prominent nucleoli and scant cytoplasm, admixed with myeloid precursors and lymphocytes (H&E, x 200). c: Mixed population of large round cells, some with indented nuclei and numerous mature and immature eosinophils (H&E, x 400). d: Large cells with irregular nuclei and small nucleoli, infiltrating into the skeletal muscle fibers (H&E, x 400). e: The cells show diffuse strong positivity for myeloperoxidase (MPO) immunostain (x 400). f: CD43 is strongly positive in immature cells (x 400). g: Cells showing diffuse strong positivity for CD 117 immunostain (x 400). h: High Ki-67 proliferative index in immature precursors (x 400).

A 38-year-old male presented with complaint of gradually progressive, left sided nasal obstruction for the last three months. Subsequently there was loss of vision in the left eye. General physical and systemic examination was unremarkable. On anterior rhinoscopy, an irregular, firm mass was seen in the left nasal cavity. Hematologic profile revealed anemia (hemoglobin = 10.4 g/dl), leucocytosis (TLC = 15,000/µl) and marked eosinophilia (absolute eosinophil count = 11,550). Computed tomography (CT) scan showed an intranasal mass involving the bilateral ethmoid sinuses with destruction of the ethmoid septae and the medial wall of the left orbit along with an extension into the inferior extraconal compartment of the left orbit and superior displacement of the left eye ball (Figure 1 (Fig. 1)). A clinical diagnosis of nasopharyngeal carcinoma was made. Biopsy from the mass showed a tumor in the sub-epithelial region invading the underlying bone. The tumor comprised variable cell population of eosinophils, neutrophils, segmented cells, and myeloid precursors admixed with large, atypical cells with hyperchromatic nuclei and prominent nucleoli (Figure 2a (Fig. 2)). Differential diagnoses included nasopharyngeal carcinoma, Hodgkin lymphoma, non-Hodgkin lymphoma (NHL), malignant melanoma, and MS. Immunohistochemistry for CK, EMA, LCA, CD3, CD20, CD30, S-100, HMB-45, CD117, MPO, and CD43 was performed for characterization of the atypical cells. Immunostaining for MPO (Figure 2e (Fig. 2)), CD43 (Figure 2f (Fig. 2)) and CD117 (Figure 2g (Fig. 2)) was strongly positive in the large cells; while all the other markers were negative. A diagnosis of MS was given. High Ki-67 labeling index was observed (Figure 2h (Fig. 2)).

Subsequent bone marrow examination showed myeloid preponderance with marked increase in mature eosinophils and presence of eosinophilic myelocytes and metamyelocytes. However, no blasts were seen.

The patient was advised chemotherapy but was lost to follow-up subsequently.

### Case 2

An 11-year-old male child presented with proptosis of the right eye for nine months. Local examination revealed a retro orbital mass. Biopsy from the mass showed large atypical cells with vesicular nuclei, prominent nucleoli, and scant cytoplasm, admixed with myeloid precursors and lymphocytes (Figure 2b (Fig. 2)). With differential diagnoses of rhabdomyosarcoma, Langerhans cell histiocytosis (LCH) and MS in mind, immunohistochemistry was applied. The atypical cells were strongly positive for MPO, CD43 and negative for CD68, S 100, CD1a, myogenin, and desmin. A diagnosis of MS was rendered and hematologic work-up was advised to rule out co-existent acute leukemia. Bone marrow examination was normal. The patient was given chemo-radiation and on follow-up of 18 months, he is disease free.

### Case 3

A 49-year-old male presented with a two week history of sudden onset weakness of left lower limb followed by altered sensorium. General physical and systemic examination was unremarkable. Neurological examination revealed decreased power in the left lower limb (grade1/5) with an extensor plantar reflex. Routine hematologic and biochemical tests were within normal limits. Magnetic resonance imaging (MRI) of the head showed a homogenously enhancing multilobulated mass in the prepontine, premedullary and left cerebellopontine angle cistern region with mass effect over the brainstem and the left cerebral hemisphere. With a clinicoradiologic diagnosis of high grade glioma, surgery with decompression of the mass was done.

Microscopic examination of the tumor tissue showed a highly cellular tumor, diffusely infiltrating the meninges and surrounding fibroadipose tissue. It consisted of mixed population of large round cells, some with indented nuclei and numerous mature and immature eosinophils (Figure 2c (Fig. 2)). Differential diagnoses included NHL, LCH, and myeloid sarcoma. Immunohistochemical stains for MPO and CD43 (Figure 2f (Fig. 2)) were strongly positive in the immature cells while a smaller number of cells were positive for CD68. Tumor cells were negative for CD1a, CD3, and CD20.

A diagnosis of MS was made and bone marrow examination was advised to rule out acute myeloid leukemia. Bone marrow examination showed a cellular marrow with normal hemopoeitic elements and eosinophilic prominence. There was no increase in blasts or presence of abnormal cells.

The patient was given chemotherapy but he expired 6 months after the diagnosis.

### Case 4

A 52-year-old male was diagnosed as a case of refractory AML seven months back and put on FLAG idarubicin. He presented with a soft tissue mass in the perineal region for the last 20 days. The swelling was nodular, firm and had a greenish hue. Biopsy was done and microscopic examination revealed a complete effacement of tissue architecture by a tumor showing large areas of necrosis. Tumor cells were large, atypical with irregular nuclei and small nucleoli and were seen infiltrating into the skeletal muscle fibers (Figure 2d (Fig. 2)). Tumor cells were strongly positive for MPO. A diagnosis of MS secondary to AML was given. 

## Discussion

MS is a localized tumor mass comprising immature granulocytic cells. These tumors are also called chloromas due to a greenish color of the tissues imparted by myeloperoxidase present in the cytoplasm of tumor cells [1]. It was first described in orbit by Burns in 1811 [3]. The pathogenesis is believed to be an aberrant expression of homing signals for the leukemic blasts on extramedullary sites as compared to bone marrow [1]. In a study by Faaij et al. in 2010, AML blasts were found to express chemokine receptors CCR5, CXCR4, CXCR7, and CX3CR1 not seen in blasts of bone marrow and peripheral blood [4].

MS can occur as a localized tissue mass in a known case of AML (3–8% of patients), before occurrence of AML (in 0.6% patients) and in association with other myeloproliferative neoplasms like blast crisis in CML and MDS. Rarely, it occurs *de novo* with normal marrow and hematologic findings as seen in three of our cases [1].

There is a predilection for males and last decades of life with a median age of 56 years. MS can involve any site of the body. Skin, soft tissues, bone, lymph nodes, GIT, and testis are affected more frequently [1], [2]. Occurrence in the head and neck region such as skull bones, orbit, and paranasal sinuses is unusual [5]. Two of our patients presented with head and neck masses, one in the orbit (Case 1) and the other in the nasal cavity (Case 2).

CNS and orbital involvement is commonly seen in the pediatric population. In the absence of overt leukemia, involvement of spinal cord is rare with only a few cases reported in the literature [6]. Thoracic spine is most commonly affected (64%) and approximately 5% cases occur in the cervical region as was seen in our case (Case 3). 

MS occurs in approximately 2–8% of patients with AML. Of these, 15–35% of the cases of MS can be seen concomitantly with AML, about 25% of the cases precede AML and up to 50% of the cases occur after the diagnosis of AML. It might as well be the initial manifestation of relapse in a patient who has been in remission [1]. The fourth case in this series developed a soft tissue mass in the perineum 8 months after being diagnosed with AML.

The most common microscopic form of myeloid sarcoma consists of a mixed population of myeloblasts, neutrophils, and neutrophil precursors. Based on the extent and degree of maturation, three major subtypes have been defined: blastic, immature, and differentiated. MS blastic type is composed primarily of myeloblasts with little evidence of maturation. The immature type is intermediate between blastic and differentiated forms and consists of myeloblasts, promyelocytes, and eosinophilic myelocytes. The differentiated or mature type is composed of promyelocytes and more mature cells with abundance of eosinophils. Myelomonocytic or pure monoblastic morphology is present in a large number of cases.

The diagnosis of MS is validated by the results of cytochemical and/or immunophenotypic analyses. The immunohistochemical panel includes CD68/KP1, antimyeloperoxidase (MPO), CD43, CD117, CD99, CD68/PG-M1, lysozyme, CD34,terminal deoxynucleotidyl transferase (TdT), CD56, CD30, glycophorin, and CD4. The combination of the above markers allows recognition of tumors with immature myeloid phenotype, as well as cases which show differentiation towards myelomonocytic, monoblastic, erythroid or megakaryocytic lineages. At times aberrant antigenic expression with cytokeratin (CK), B- or T-cell markers is noted. In 55% of the cases chromosomal aberrations are known to occur which can be confirmed by FISH and/or cytogenetics. These include monosomy 7, trisomy 8, *MLL* rearrangement, inv(16), trisomy 4, monosomy 16, 16q-, 5q-, 20q- and trisomy 11 [7].

In the absence of peripheral blood and bone marrow involvement MS can be misdiagnosed as a number of other neoplastic diseases depending on the site of involvement. The major differential diagnosis of MS is lymphoma including lymphoblastic lymphoma, Burkitt lymphoma, and diffuse large B-cell lymphoma. Other less common differentials are soft tissue sarcomas, small round cell tumors (especially in children) and undifferentiated carcinoma. It is important to distinguish between these entities by cytochemical and immunohistochemical stains for prompt recognition and institution of appropriate treatment [8].

The prognosis of MS depends upon the initial context in which it appears. Most patients with isolated MS progress to AML within months of diagnosis. Cases that progress to AML seldom survive more than a year [9].

Patients with primary myeloid sarcoma should be treated as AML even in the absence of clinically detectable leukemia. The treatment modalities include induction/intensification therapy similar to those with AML. The role of radiotherapy is also described by some authors [10].

## Conclusion

MS is an uncommon tumor and can occur in unusual locations with variable presentation as seen in the present series. Therefore, it should be considered in the differential diagnosis of a soft tissue mass regardless of the presence/absence of leukemia. It is important to make a correct diagnosis by using the armamentarium of morphology, histochemistry, and immunohistochemistry collectively for prompt institution of therapy.

## Notes

### Competing interests

The authors declare that they have no competing interests.

## Figures and Tables

**Table 1:**
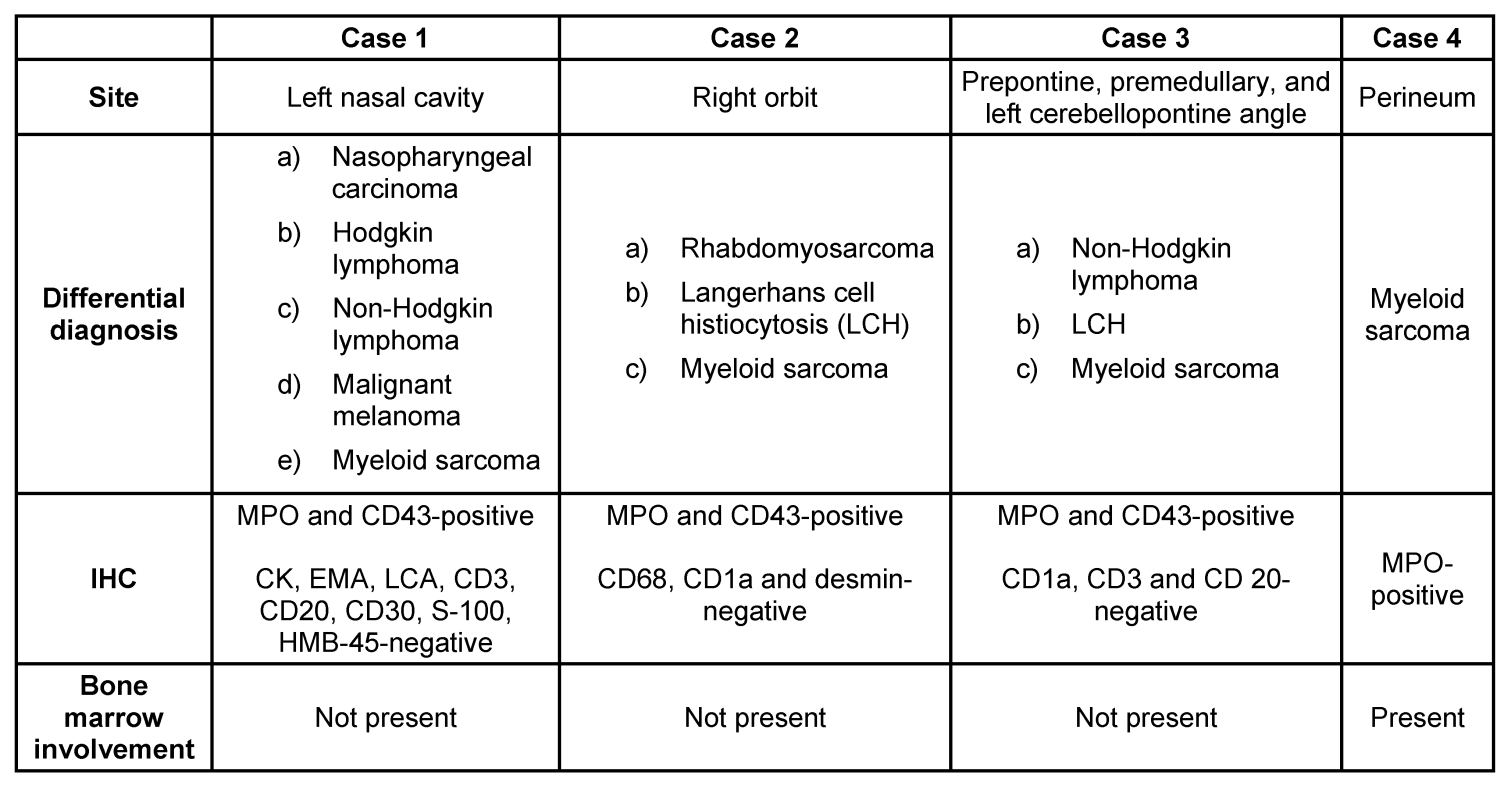
Clinicopathologic features of the four cases
